# A Rabbit Monoclonal Antibody against the Antiviral and Cancer Genomic DNA Mutating Enzyme APOBEC3B

**DOI:** 10.3390/antib8030047

**Published:** 2019-09-10

**Authors:** William L. Brown, Emily K. Law, Prokopios P. Argyris, Michael A. Carpenter, Rena Levin-Klein, Alison N. Ranum, Amy M. Molan, Colleen L. Forster, Brett D. Anderson, Lela Lackey, Reuben S. Harris

**Affiliations:** 1 Department of Biochemistry, Molecular Biology and Biophysics, University of Minnesota, Minneapolis, MN 55455, USA; 2 Masonic Cancer Center, University of Minnesota, Minneapolis, MN 55455, USA; 3 Institute for Molecular Virology, University of Minnesota, Minneapolis, MN 55455, USA; 4 Center for Genome Engineering, University of Minnesota, Minneapolis, MN 55455, USA; 5 Howard Hughes Medical Institute, University of Minnesota, Minneapolis, MN 55455, USA; 6 Division of Oral and Maxillofacial Pathology, School of Dentistry, University of Minnesota, Minneapolis, MN 55455, USA; 7 Clinical and Translational Science Institute, University of Minnesota, Minneapolis, MN 55455, USA; 8 Department of Biology, University of North Carolina, Chapel Hill, NC 27599, USA

**Keywords:** APOBEC3B, cancer biomarker, DNA cytosine deaminase, IHC assay, rabbit monoclonal antibody

## Abstract

The DNA cytosine deaminase APOBEC3B (A3B) is normally an antiviral factor in the innate immune response. However, A3B has been implicated in cancer mutagenesis, particularly in solid tumors of the bladder, breast, cervix, head/neck, and lung. Here, we report data on the generation and characterization of a rabbit monoclonal antibody (mAb) for human A3B. One mAb, 5210-87-13, demonstrates utility in multiple applications, including ELISA, immunoblot, immunofluorescence microscopy, and immunohistochemistry. In head-to-head tests with commercial reagents, 5210-87-13 was the only rabbit monoclonal suitable for detecting native A3B and for immunohistochemical quantification of A3B in tumor tissues. This novel mAb has the potential to enable a wide range of fundamental and clinical studies on A3B in human biology and disease.

## 1. Introduction

The APOBEC3 family of single-stranded DNA cytosine deaminases constitutes a vital arm of the innate immune response, which serves to restrict the replication of viruses and transposable elements [1,2,3,4,5]. A wide range of parasitic elements have been shown to be susceptible to restriction and/or mutation by APOBEC3 enzymes, including retroviruses and retrotransposons with obligate single-stranded DNA replication intermediates, hepadnaviruses such as Hepatitis B virus (HBV), small DNA tumor viruses such as Human Papillomavirus (HPV), polyomaviruses (BK-PyV/JC-PyV), and large DNA viruses such as the γ-herpesviruses Epstein-Barr Virus (EBV) and Kaposi’s Sarcoma-associated Herpesvirus (KSHV) (see, e.g., [6,7,8,9,10,11,12,13,14,15,16]). Thus, the human APOBEC3 enzymes have become an important focus of research in multiple areas of virology.

APOBEC has also emerged as major endogenous source of mutation in a wide variety of different cancers [3,5,17,18,19,20]. APOBEC mutations in cancer are defined as C-to-T and C-to-G base substitutions in 5′-TCA/T trinucleotide motifs. This APOBEC mutation signature is evident in over half of all human cancer types, and it is one of the dominant etiologies in bladder, breast, cervix, head/neck, and lung cancers [21,22,23,24]. APOBEC3B (A3B) is the leading candidate for APOBEC mutagenesis in cancer, due to several independent lines of evidence including overexpression in tumors and cancer cell lines, nuclear localization, modulation by tumor-promoting viruses (including HPV and EBV), and associations with poor clinical outcomes (see, e.g., [11,16,22,25,26,27,28,29,30,31,32,33,34,35]). APOBEC3H (A3H) may also contribute to cancer mutagenesis, particularly in populations that lack A3B due to a naturally occurring deletion allele [36,37]. APOBEC3A (A3A) has also been implicated through a number of lines of investigation; however, studies on the endogenous enzyme have not yet been carried out in model systems, due to low/no expression [23,38,39,40,41,42,43,44,45,46]. Ongoing research by many groups is likely to clarify the relative contributions of each APOBEC enzyme in the mutagenesis of different tumor types, and new technologies and reagents including validated monoclonal antibodies (mAbs) will have essential roles in this process.

A problem in studying human APOBEC3 enzymes in virology and cancer biology is the fact that human cells have the potential to express up to seven different family members: A3A, A3B, and A3H, as described above, as well as APOBEC3C (A3C), APOBEC3D (A3D), APOBEC3F (A3F), and APOBEC3G (A3G). Moreover, due to multiple primate-specific gene duplication and diversification events, several of the present-day human A3 enzymes share high levels of amino acid identity [47,48]. For instance, A3A and the catalytic domain of A3B are over 90% identical, and each of these proteins shares over 65% identity with the catalytic domain of A3G. Thus, it has been a major challenge to develop mAbs for distinguishing between these enzymes in various immunoassays.

Here, we report the development of a rabbit mAb, called 5210-87-13, for human A3B. This reagent has proven to be effective in a variety of different immunoassays, including enzyme-linked immunosorbent assay (ELISA), immunoblot (IB), immunofluorescent microscopy (IF), flow cytometry (FLOW), and immunohistochemistry (IHC). Importantly, head-to-head comparisons with commercial rabbit mAbs demonstrated that only 5210-87-13 was able to effectively stain A3B in the nuclei of tumor cells in formalin-fixed and paraffin-embedded (FFPE) tumor tissue sections. This advance opens the door to rigorously testing APOBEC3B as a cancer biomarker through protein-level association studies between APOBEC3B IHC levels and clinical outcomes including treatment responses and survival.

## 2. Materials and Methods

### 2.1. Cell Lines

HeLa and 293T cells were grown in Dulbecco’s Modified Eagle Medium (DMEM), while U2OS cells were cultured in McCoy’s 5A medium supplemented with 10% fetal bovine serum (FBS, Thermo Fisher Scientific, Waltham, MA, USA) and 1% penicillin-streptomycin (P/S, Thermo Fisher Scientific). T-REx-293 is a derivative of 293T, engineered to constitutively expresses the tetracycline repressor [25,49,50]. THP18 [51] was derived from a single-cell subclone of the monocyte cell line THP-1 (ATCC TIB-202, American Type Culture Collection, Manassas, VA, USA) and maintained in RPMI-1640 growth medium supplemented with 10% FBS. MCF-7L and its *A3B*-null derivative have been described [33,36] and were maintained in Improved Minimal Essential Medium (IMEM) supplemented with 5% FBS, 1% P/S, non-essential amino acids (0.1 mM, Life Technologies, Grand Island, NY, USA), and insulin (11.25 nM, MilliporeSigma, Burlington, MA, USA). The multiple myeloma cell lines NCI-H929 and JJN3 were kind gifts from Brian Van Ness (University of Minnesota Twin Cities, Minneapolis, MN, USA) and were grown in RPMI-1640 supplemented with 15% FBS, 1% P/S, and 50 µM β-mercaptoethanol. Primary Effusion Lymphoma (PEL) cell lines JSC-1 and BC-3 were kind gifts from Bill Sugden (University of Wisconsin Madison, Madison, WI, USA) and grown in RPMI-1640 supplemented with 10% FBS and 1% P/S.

### 2.2. Plasmids

The A3-HA plasmid set has been described [7]. The A3B-eGFP expression construct and the shRNA expression constructs have also been reported [25,52,53].

### 2.3. Generation of A3A and A3A/B Knockout Cell Lines

*A3A* and *A3A/B* deletion THP18 cell lines and *A3B* deletion JJN3 cells were engineered by co-transduction with plentiCRISPR, expressing the gRNA sequences flanking the *A3A* gene (CCTGGACAAGCGACATACCG and AATGCACCATGTCTCCCCTC), both the *A3A* and *A3B* genes (CCTGGACAAGCGACATACCG and ACAACACCCTCGCCCCATGA), or the *A3B* gene alone (AATGCACCATGTCTCCCCTC and ACAACACCCTCGCCCCATGA). As a control, the parental THP18 cell line was transduced in parallel with an unrelated gRNA (GGCGACCACCGCCGCCATCT). Three days following transduction, cells were treated with 1 µg/mL puromycin for 3 days and seeded for single cell cloning in 96 well plates, 1 week later. Deletion mutant lines were identified by PCR using primers amplifying unique segments within the *A3B* gene [54] (TTGGTGCTGCCCCCTC and TAGAGACTGAGGCCCAT) and/or the *A3A* gene (CCTCCTCTGGTCTTTTCCCT and GAAACCACAAGTACAATCCGG) and confirmed by qPCR and immunoblots.

### 2.4. Hybridoma Generation

The full protein sequences of the seven human APOBEC3 (A3) enzymes were obtained from GenBank (A3A GenBank: AAI26417.1; A3B GenBank: AAW31743.1; A3C GenBank: AAH11739.1; A3D GenBank: AIC57731.1; A3F GenBank: AAZ38720.1; A3G GenBank: AAZ38722.1; A3H GenBank: ACK77774.1). ClustalW was used to identify regions unique to A3B. Residues 354–382, PFQPWDGLEEHSQALSGRLRAILQNQGN, were used to create a peptide immunogen (Epitomics, Cambridge, MA, USA).

Two rabbits were given three injections using Keyhole Limpet Hemocyanin (KLH)-conjugated peptide, then two further injections with Ovalbumin (OVA)-conjugated peptide over the course of 10–12 weeks (Epitomics). Test bleeds from the rabbits were screened at UMN for anti-A3B expression by immunoblot with lysates from 293T cells expressing A3 proteins tagged with the human influenza Hemagglutinin (HA) epitope. The bleeds were further screened at the University of Minnesota (UMN) by immunofluorescence microscopy (IF) of HeLa cells expressing the A3B-eGFP protein. Rabbits showing positive anti-A3B immune responses were selected for a final immunization boost before their spleens were harvested for B-cell isolation and hybridoma production. Hybridoma fusions of 240E-W cells with lymphocytes from the selected rabbits were performed by Epitomics in 40 × 96-well plates. Cell supernatants were screened at UMN by A3B ELISA and the strongest positive hybridoma pools were subcloned by standard limiting dilution to generate monoclonal hybridoma cell lines. Hybridoma 5210-87-13 was expanded to 1 L and then switched to a serum-free medium for one week. This medium was clarified by centrifugation to remove cells and then passed over a Protein A column to bind mAb. The resulting mAb was eluted in glycine pH 2.5, dialyzed into phosphate buffered saline (PBS), aliquoted into sealed tubes, and stored at −80 °C.

### 2.5. ELISA

Standard ELISA screening with recombinant A3Bctd (A3BctdQM∆loop3) [55] was used to monitor anti-peptide immune responses in rabbits and to identify single-clone hybridomas which expressed anti-A3B antibodies. Recombinant A3Bctd (20 ng/well) was immobilized on 96-well ELISA plates, blocked with 3% BSA in PBS, and then incubated with undiluted cell-free media supernatant from candidate hybridomas. Supernatants from cells that did not express A3B binding activity were used as negative controls. The positive control was a polyclonal rabbit anti-human A3G antibody (NIH AIDS Reagent Program, 10201) which cross-reacts with human A3B. Binding was detected with a goat anti-rabbit HRP secondary antibody (Jackson ImmunoResearch Laboratories Inc., West Grove, PA, USA, 1:5000), visualized with tetramethylbenzidine (TMB), and quantified by spectroscopy at 450 nm using a BioTek Synergy H1Microplate reader.

### 2.6. Immunoblots

Cell lysates from 293T cells, transiently transfected with each HA-tagged A3 protein or the expression vector alone, were resolved by 12% polyacrylamide gel electrophoresis (SDS-PAGE) and transferred to polyvinylidene difloride membrane (PVDF, MilliporeSigma). Membranes were probed with the test bleeds (1:1000), cell-free supernatants from each hybridoma cell line (1:3), purified 5210-87-13 mAb (1:2000), anti-HA (C29F4, #3724, Cell Signaling Technology, Danvers, MA, USA, 1:1000), or anti-tubulin (MMS-407R, Covance, Emeryville, CA, USA, 1:20,000) in 50% BLOK (MilliporeSigma) and 0.1% Tween 20 in PBS. The membranes were then incubated with secondary antibody, either goat anti-rabbit-HRP (1:5000 Jackson ImmunoResearch Laboratories Inc.), anti-rabbit IgG IR800CW (Odyssey 926-32211, 1:20,000, LI-COR, Lincoln, NE, USA), or anti-mouse IgG IR800CW (Odyssey 827-08364, 1:20,000) in 50% BLOK (MilliporeSigma) and 0.1% Tween 20 in PBS. A subset of immunoblots used Abcam 1184990 (1:2500, Cambridge, MA, USA) and Proteintech 14559-1-ap (1:500, Proteintech Group Inc., Rosemont, IL, USA) mAbs diluted in PBS, supplemented with 5% milk protein and 0.1% Tween 20. Signals were detected with HyGlo (Thomas Scientific, Swedesboro, NJ, USA) on film or imaged using LiCor.

### 2.7. Immunofluorescence Microscopy

HeLa cells (2 × 10^5^) were transfected with 200 ng of an A3B-eGFP expression construct. T-REx-293-A3B-eGFP cells (2 × 10^5^) were induced with 1 µg/mL doxycycline. After 24 h, cells were washed twice with PBS, fixed in 4% paraformaldehyde (PFA) for 30 min, washed twice with PBS, blocked (5% goat serum, 1% BSA, 0.2% TritonX-100) for 1 h at 4 °C, and then incubated overnight at room temperature with primary antibody in blocking buffer. Supernatant from 5210-87-13 hybridoma cell culture was used at 1:5 dilution and purified 5210-87-13 mAb was used at 1:250 dilution in blocking buffer. Endogenous A3B was detected using purified 5210-87-13 (1:25), Abcam 184990 (1:50), or Proteintech 14559-1-ap (1:25). Cells were washed twice with 1× PBS and incubated in secondary antibody, anti-rabbit-TRITC (1:500 Jackson ImmunoResearch Laboratories Inc. 111095144), or anti-rabbit Alexa Fluor 594 1:500 in blocking buffer for 1 h at room temperature. Following two washes with PBS, the nuclei were strained with 0.1% Hoescht for 15 min at room temperature. Following two washes with 1× PBS, slides were mounted in 50% glycerol in 1× PBS. Images were taken at 60× magnification, with a 1 s tetramethylrhodamine (TRITC) exposure time (to normalize), cropped to 900 × 900 pixels, and scaled down to 10% size.

### 2.8. Immunohistochemistry

Human tissues were obtained using a protocol reviewed by the University of Minnesota Institutional Review Board. All formalin-fixed, paraffin-embedded (FFPE) specimens were derived from incisionally or excisionally biopsied cervical, breast, bladder, and head/neck carcinomas. Tissues were sectioned at 4 µm, mounted on positively charged adhesive slides and allowed to air-dry for at least 24 h. To deparaffinize and rehydrate the samples, slides were baked in a 65 °C oven for 20 min, washed 3 times with CitrisolvTM (Decon Labs, King of Prussia, PA, USA. #1601) or xylene for 5 min each, soaked in graded alcohols (100% × 2, 95% and 80% for 3 min each), and then rinsed in running water for at least 5 min. Epitope retrieval was performed using Reveal Decloaker (BioCare Medical, Pacheco, CA, USA, # RV1000M) in a steamer for 35 min, followed by a 20 min “cool-down” period. Then, the slides were rinsed with running tap water for 5 min and transferred to Tris-buffered saline with 0.1% Tween 20 (TBST) for 5 min. Endogenous peroxidase activity was quenched by placing the slides in 3% H_2_O_2_ in TBST for 10 min at room temperature (RT), followed by a 5 min rinse under running water. To block non-specific binding of the primary antibody, sections were covered with Background Sniper (BioCare Medical, # BS966MM) for 15 min at RT. After blocking, serial sections of each tumor were incubated overnight at 4 °C with 5210-87-13 mAb diluted (1:300–1:1000) in 10% Sniper in TBST. A subset of IHC experiments used Abcam 184990 or Proteintech 14559-1-ap rabbit mAbs diluted 1:1000 and 1:2000, respectively. Immunostaining against cells of lymphocytic and monocytic lineage was performed using anti-CD3 (clone SP1, Abcam, 1:400) and anti-CD14 (clone EPR3653, MilliporeSigma, 1:100) antibodies, respectively.

Following overnight incubation with primary antibody, sections were rinsed in TBST for 5 min, and completely covered with post-primary rabbit anti-mouse IgG (Novolink, # RE7260-K, Leica Biosystems, Buffalo Grove, IL, USA,) for 30 min at RT. The slides were then drip-dried, transferred to TBST for 5 min, and incubated with anti-rabbit poly-HRP-IgG (Leica Biosystems, Novolink Polymer, #RE7260-K) for 30 min at RT. The reaction product was developed using the Novolink DAB substrate kit (Leica Biosystems, # RE7230-K) at RT for 5 min, rinsed in tap water for 5 min, counterstained in Mayer’s hematoxylin solution (Electron Microscopy Sciences, Hatfield, PA, USA; #26252-01) for up to 5 min, dehydrated in graded alcohols and CitrisolvTM or xylene, and cover-slipped using Permount mounting media. The immunohistochemical stains were scanned at 40× magnification and visualized using the Aperio ScanScope XT system (Leica Biosystems).

### 2.9. Cell Microarray (CMA) and Histoscore Determination

1.0 × 10^6^ THP18 (WT), THP18-ΔA3A, and THP18-ΔA3A/B cells were seeded in 6-well plates, allowed to grow for 24 h, and were then induced with 1 µg/mL lipopolysaccharide (LPS) (MilliporeSigma, #L2654) and 300 U/mL of Interferon-alpha (IFN-α) (R&D Systems Inc., Minneapolis, MN, USA, #11200-1) for 24 h. The 293T and inducible T-REx-293-A3B-eGFP cells were also included in the CMA, with the latter induced for 24 h with 1µg/µL doxycycline. Cell buttons were prepared as follows: Trypsinization, centrifugation, and supernatant removals were followed by 3 washes with PBS. Following the last wash, cells were resuspended in 10% formalin for 15 min. After fixation, cells were rinsed 3× with PBS, mixed with Histogel (Thermo Fisher Scientific, #HG-4000-012) at 65 °C, and allowed to solidify at RT for approximately 10 min. The resulting cell blocks were placed in Histosette II cassettes, dehydrated, and embedded in paraffin.

Immunohistochemical staining of the CMA was performed as above for tissues. Nuclear A3B immunoreactivity was visualized with the Aperio ScanScope XT (Leica Biosystems) and quantified using the Aperio Nuclear Algorithm. Histoscore (H-Score) was calculated using the formula (3+) × 3 + (2+) × 2 + (1+) × 1, as described earlier [56,57].

## 3. Results

### 3.1. Epitope Design, Hybridoma Generation, and Immunoblot Data

**Figure 1:**
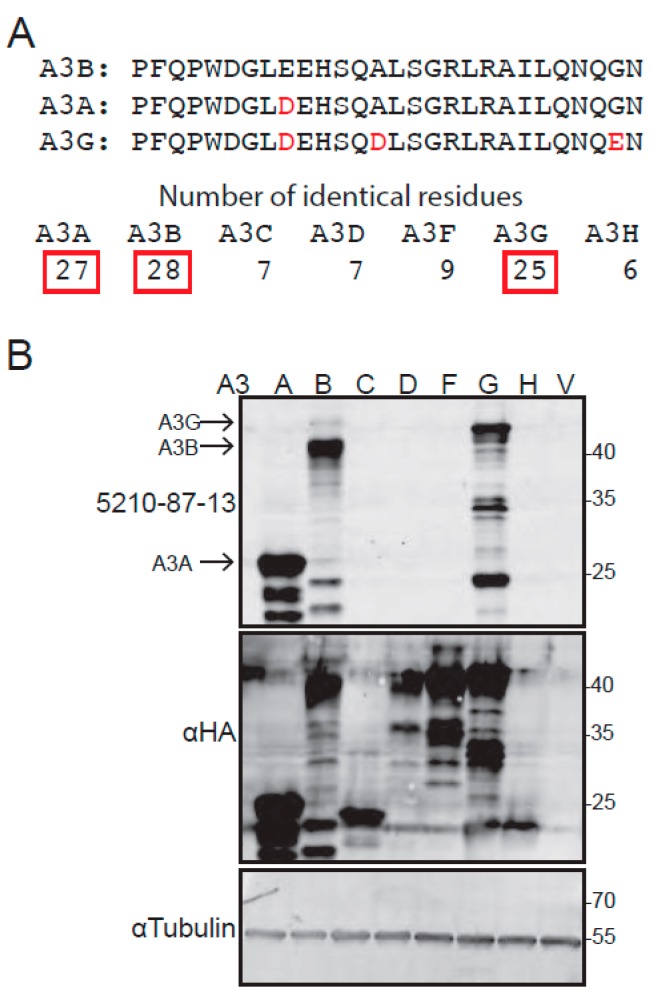
Peptide selection and anti-APOBEC3 reactivity of the 5210-87-13 mAb by immunoblot: (**A**) Comparison of the C-terminal region of A3B with the corresponding regions of A3A and A3G; and (**B**) anti-A3 reactivity of 5210-87-13, with anti-HA and anti-Tubulin blots as expression and loading controls, respectively. Faster migrating bands are likely degradation products, as minimal background is observed in lanes with A3C, A3D, A3F, A3H, and the vector control (V).

The full protein sequences of the seven human APOBEC3 enzymes were obtained from GenBank. The alignment feature of ClustalW was used to identify regions unique to A3B, and a C-terminal region was selected for synthesis of a peptide immunogen, corresponding to residues 354–382. Although this 28-residue sequence is unique to A3B, homology with related family members was unavoidable and the corresponding regions of A3A and A3G still shared 27/28 and 25/28 residues, respectively (Figure 1A). The selection of this peptide was also supported by the knowledge that the corresponding C-terminal peptide of A3G is immunogenic in rabbits [58,59].

The A3B peptide 354–382 was used to immunize two rabbits over a period of 10–12 weeks. The initial immunization occurred through three injections of KLH-conjugated peptide, and an additional boost was done through two injections of OVA-conjugated peptide. Throughout the immunization protocol, sera were assessed for anti-A3B activity by ELISA, IB, and IF microscopy assays. Both rabbits (5210, 5211) showed positive anti-A3B immune responses and were given a final antigen boost one week before their spleens were harvested for B-cell isolation and hybridoma production.

Splenic B lymphocytes were fused with the rabbit plasmacytoma cell line 240E-W to create hybridoma candidates (40 × 96-well plates). Standard ELISA assays were used to screen culture supernatants from 3840 candidate wells for immunoreactivity against recombinant A3B C-terminal domain (A3BctdQM∆loop3) [55]. Positive candidates were expanded and supernatants were retested by ELISA and further screened by IB and IF (data not shown). Finally, the strongest ELISA-positive A3B-reactive candidates were subcloned by limiting dilution to create monoclonal hybridomas for further characterization.

Sera from expanded monoclonal hybridomas were, then, tested for specificity against human A3B by immunoblotting. A3B-low 293T cells (quantification reported in supplemental data of [16]) were transiently transfected with HA-tagged A3 expression constructs (A3A-HA, A3B-HA, A3C-HA, A3D-HA, A3F-HA, or A3G-HA) or the empty expression vector alone, as a negative control. After 48 h incubation for protein expression, whole cell extracts were prepared, fractionated by SDS-PAGE, and probed using supernatants from each candidate hybridoma. Several ELISA-positive and IB-positive mAb candidates were obtained; all showed varying degrees of reactivity against A3B, as expected, but also varying degrees of reactivity against A3A and A3G. The hybridoma clone 5210-87-13 was selected for in-depth characterization, as it reacted most strongly and reproducibly with A3B (upper panel, Figure 1B). Control anti-HA immunoblots confirmed expression of each A3-HA construct, and control anti-Tubulin immunoblots showed similar protein loading in each lane (lower panels, Figure 1B).

### 3.2. Immunofluorescent Microscopy Results Using the 5210-87-13 mAb

**Figure 2:**
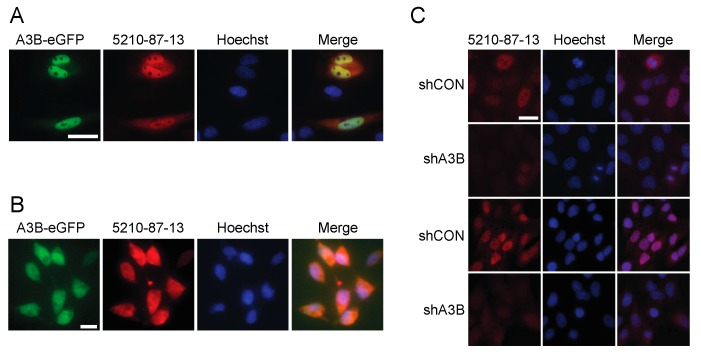
Immunofluorescent (IF) microscopy results for the 5210-87-13 mAb: (**A**) IF microscopy images of HeLa expressing A3B-eGFP and stained with the 5210-87-13 mAb (scale bar is 10 µm); (**B**) IF microscopy images of doxycycline-induced T-REx-293-A3B-eGFP cells stained with the 5210-87-13 mAb (scale bar is 10 µm); and (**C**) IF microscopy images of 5210-87-13 stained U2OS cells expressing a control shRNA (shCON) or shA3B (scale bar is 10 µm). Data from two independent experiments are provided for each condition, to show the reproducibility of this result.

Prior studies have shown that the bulk of cellular A3B is nuclear, due to an active import mechanism (see, e.g., [25,52,53,60]). To determine whether the 5210-87-13 mAb recognizes nuclear-localized A3B, HeLa cells were transfected with an A3B-eGFP expression construct and subjected to IF microscopy. A3B-eGFP-expressing HeLa cells showed a strong green fluorescence signal in the nucleus (Figure 2A). Staining the same cells with the 5210-87-13 mAb followed by a secondary goat anti-rabbit-IgG-TRITC resulted in a red signal from the same nuclear regions (Figure 2A). Signal co-localization was confirmed in the merged image with staining of the entire nucleus except nucleoli. Similar results were obtained with T-REx-293-A3B-eGFP cells treated with doxycycline to induce A3B-eGFP expression (Figure 2B), except that a broader cell-wide co-localization was observed; likely due to the combined effects of gross over-expression and a previously reported nuclear import defect in 293 and derivative cell lines [52,61].

Next, IF microscopy was used to ask whether the 5210-87-13 mAb could detect endogenous A3B. U2OS osteosarcoma cells were transduced with either a retroviral construct expressing a shRNA against *A3B* to knockdown endogenous *A3B* gene expression, or with a construct expressing a scrambled shRNA to generate a control cell population. A3B-depleted and control U2OS cells were then stained, as above, with 5210-87-13 mAb (primary) and anti-rabbit-IgG-TRITC (secondary) and subjected to fluorescence microscopy. A3B-depleted cells were negative or stained poorly, while the control population with endogenous A3B showed a prominent red nuclear fluorescence signal, largely coincident with Hoescht (results from two independent experiments shown in Figure 2C). Similar IF microscopy results have also been reported recently for endogenous A3B in select B lymphocyte-derived cell lines [16]. As previously reported, A3B is excluded from chromatin during mitosis and cell-wide [52,53], then rapidly reimported into the nucleus (e.g., top cell in the shCON image in Figure 2C). Taken together, these immunofluorescent microscopy data show that the 5210-87-13 mAb is capable of labeling both transiently and endogenously expressed native A3B in different cell types.

### 3.3. Immunoblot Results Using Cancer Cell Lines and the 5210-87-13 Anti-A3B mAb

**Figure 3:**
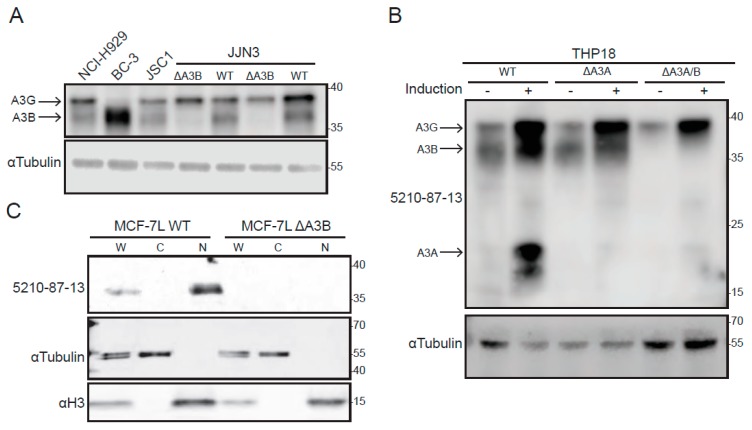
Endogenous A3B detection by 5210-87-13 mAb immunoblotting: (**A**,**B**) Immunoblots of whole cell lysates from the indicated cell lines probed with the 5210-87-13 mAb and anti-Tubulin as a loading control; and (**C**) immunoblots of whole-cell (W), cytoplasmic (C), and nuclear (N) fractions from the indicated cell lines probed with the 5210-87-13 mAb and with anti-Tubulin and anti-histone H3 as nuclear and cytoplasmic loading controls, respectively.

Given strong A3B overexpression in various tumor types (see Section 1), cancer cell lines were selected for additional 5210-87-13 IB validation studies. First, two multiple myeloma cell lines (NCI-H929 and JJN3) and two primary effusion lymphoma cell lines (BC-3 and JSC-1) were tested, due to RNAseq data indicating relatively high *A3B* expression [62]. These immunoblots showed strong reactivity around 36 kDa, relative to the molecular weight size marker, which is the position where endogenous A3B typically migrates (Figure 3A). Endogenous A3G migrates slightly slower with, for instance, BC-3 expressing high A3B and low A3G and the other lines expressing similar levels of both proteins. The identity of the 36 kDa A3B band was confirmed in JJN3 cells through two independent CRISPR knockouts (Figure 3A). It is important to note that both A3B and A3G migrate faster in SDS-PAGE gels than their actual molecular weights (45.9 and 46.5 kDa, respectively) and that separation can be facilitated by gel type and run duration (see Section 2).

Additional validation studies were done using THP18, a subclone of the monocytic cell line THP1, and derivative lines engineered by CRISPR to lack either *A3A* or both *A3A* and *A3B* (Figure 3B). THP1 is known to express A3B and A3G in normal growth medium and, following interferon-α (IFN-α) treatment, induced levels of A3A [49,59,63]. These lines were expanded in normal growth medium, treated with IFN-α and LPS, processed into whole-cell extracts, and analyzed by immunoblotting with the 5210-87-13 mAb (Figure 3B). The resulting immunoblot showed that A3A, A3B, and A3G are all highly expressed under these conditions, with A3A undergoing the most dramatic change, from undetectable to a robust visible signal (two bands, due to alternative methionine codons for translation initiation, as described in [49,59,63]). Importantly, A3A expression was abolished in the *A3A* knockout, and both A3A and A3B expression were abolished in the double knockout (THP18 *∆A3A/B*, Figure 3B).

Last, nuclear and cytoplasmic fractions from a previously described MCF-7L breast adenocarcinoma cell line and a derivative engineered by CRISPR to lack A3B [36] were analyzed by immunoblotting with the 5210-87-13 mAb (Figure 3C). This experiment showed, as expected, that A3B is detectable in whole-cell extracts, absent from cytoplasmic fractions, enriched in nuclear lysates and, importantly, completely undetectable in engineered *A3B* knockout cells. As controls, tubulin is enriched in cytoplasmic fractions and histone H3 in nuclear fractions. No cross-reactivity with A3G or A3A was observed in MCF-7L, as this line does not express significant levels of the mRNAs that encode these proteins [36].

### 3.4. IHC Detection of A3B in Cell Lines Using the 5210-87-13 mAb

**Figure 4:**
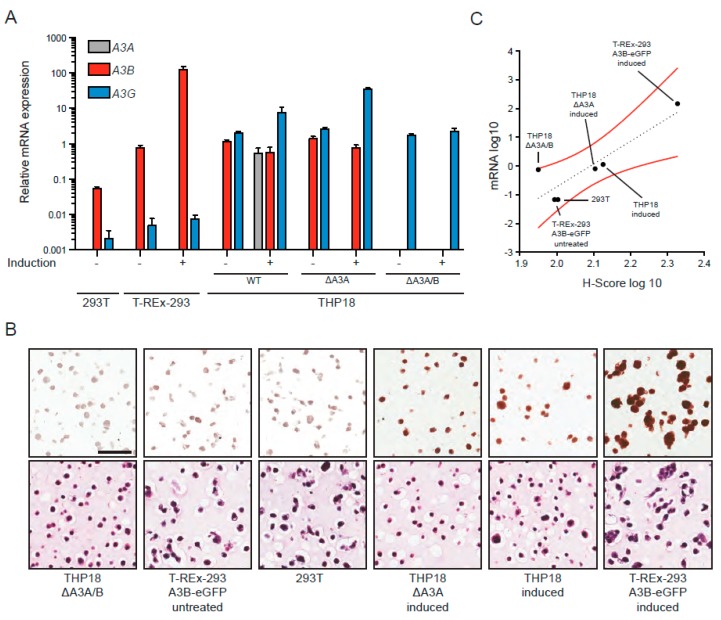
*A3B* mRNA levels correlate with IHC histo-score (H-score) using the 5210-87-13 mAb: (**A**) Assessment of *A3A*, *A3B* and *A3G* mRNA levels by RTqPCR in the indicated cell lines; (**B**) representative IHC images from 5210-87-13 staining of the indicated cell lines in the context of a cell microarray (CMA) (top row), along with corresponding hematoxylin photomicrographs (bottom row). Scale bar is 60 µm; and (**C**) dot plot showing the positive correlations between *A3B* mRNA levels and IHC H-scores from 5210-87-13 mAb nuclear straining of the indicated cell lines (H-score; *R*^2^ = 0.8, Pearson correlation coefficient = 0.9).

Next, we wanted to evaluate the efficacy of the 5210-87-13 mAb in IHC experiments using a custom-built cell microarray. As described above, we have both identified and purposefully engineered systems to express the broadest possible range of A3B protein from absolute zero (*A3B* knockout cell lines) to massive overexpression (T-REx-293-A3B-eGFP cells treated with doxycycline). Two isogenic sets of cell lines were selected for these IHC experiments: (1) 293T and T-REx-293-A3B-eGFP minus/plus doxycycline induction, and (2) THP18, THP18 *∆A3A* and THP18 *∆A3A/B* minus/plus IFN-α/LPS induction. In all instances, cell lines were grown under normal conditions, treated to induce *A3B* or *A3A/B/G* expression, and then split into aliquots for mRNA quantification by RTqPCR using established assays [25,36,63,64] and for protein detection by IHC using the 5210-87-13 mAb. The RTqPCR results largely mirrored the immunoblot data described previously for the T-REx-293-A3B-eGFP system [25,49,50], and as shown above in Figure 3B for the THP18 cell line and its *A3A*-null and *A3A/B*-null derivatives. *A3A* mRNA was undetectable in the 293T cells (regardless of treatment), induced by three orders of magnitude in INF-α/LPS treated THP18 and, of course, was absent from *A3A*-null and *A3A/B*-null derivatives (Figure 4A). *A3B* mRNA was weakly expressed in 293T and modestly expressed in untreated T-REx-293-A3B-eGFP cells (due to leaky *A3B-eGFP* expression); in contrast, doxycycline treatment induced *A3B* mRNA levels by two orders of magnitude (Figure 4A). *A3B* mRNA levels decreased modestly following IFN-α/LPS treatment, despite clear increases in protein levels in the IB experiments, further emphasizing the need for protein-level quantification (compare data in Figure 4A and Figure 3B). In comparison, *A3G* mRNA was expressed weakly in the 293T lines (undetectable by IB) and induced by IFN-α/LPS treatment in THP18 and most derivatives, consistent with the IB results (Figure 4A and Figure 3B).

In the IHC experiments, doxycycline-induced T-REx-293-A3B-eGFP cells showed the strongest nuclear A3B immunoreactivity in almost 100% of the examined cells (far right images in Figure 4B). On the other end of the signal intensity scale, the *A3A/B*-null THP18 cell line exhibited sporadic and weak immunostaining with the 5210-87-13 mAb, suggesting that the potentially cross-reactive epitope in A3G may be either blocked or shielded under these experimental conditions (far left images in Figure 4B). Consistent with this interpretation, IFN-α/LPS treated THP18 and *A3A*-null derivatives exhibited the second and third highest (strong to moderate) nuclear A3B immunoreactivities, respectively. In comparison, untreated parental 293T and T-REx-293-A3B-eGFP cells exhibited sporadic and weak immunostaining. Despite likely cross-reactivity with A3A and little/no cross reactivity under these conditions with A3G, a strong positive correlation was evident between *A3B* mRNA levels and the 5210-87-13 IHC H-score (*R*^2^ = 0.8, Pearson correlation coefficient = 0.9; Figure 4C).

### 3.5. IHC Detection of Endogenous A3B in Tumor Tissue Using the 5210-87-13 mAb

**Figure 5:**
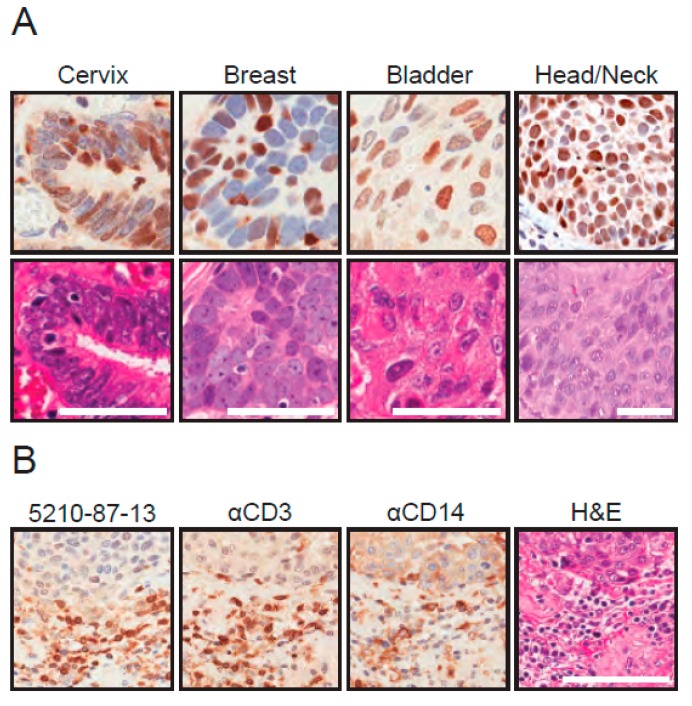
IHC detection of endogenous A3B protein in tumors using the 5210-87-13 mAb: (**A**) 5210-87-13 mAb staining of the indicated FFPE tumor specimens prescreened by RTqPCR for high *A3B* mRNA levels (top row). A hematoxylin-stained serial section is shown below each tumor (scale bars are 50 µm). (**B**) 5210-87-13 mAb, anti-CD3, and anti-CD14 staining of a representative FFPE tumor specimen with inflammation and infiltrating immune cells (scale bar is 100 µm). See text for details.

Next, FFPE tissues were stained with the 5210-87-13 mAb to determine whether the cell-based IHC results may extend to primary tumor specimens. Tumor tissues were chosen to reflect cancers in which *A3B* mRNA overexpression has been reported, specifically cancers of the cervix, breast, bladder, and head/neck [25]. In many instances, the 5210-87-13 mAb specifically and clearly stained the nuclear compartment of tumor cells, but not the surrounding non-tumor cells and connective tissues (Figure 5A and data not shown). Interestingly, intra-tumor heterogeneity was observed with respect to both distribution and intensity (e.g., a strong A3B-positive tumor cell may be next to an A3B-negative tumor cell). This heterogeneity may be related to cell-cycle regulation [11,65], but a definitive molecular explanation is not available at this time; again, underscoring the need for protein-level detection methods for A3B in cancer.

In addition, in a subset of tumors with evidence of inflammation, the surrounding connective tissue stroma was infiltrated by a mixed population of CD3-positive and CD14-positive inflammatory cells (Figure 5B). CD3 and CD14 are diagnostic markers for T-lymphocytic and monocytic lineage cells, respectively. In these cell types, the strongest 5210-87-13 mAb reactivity was noticed in the cytoplasmic compartment, where A3G and A3A are known to localize [7,49,66,67,68]. Thus, these observations, combined, indicate that 5210-87-13 mAb has the capacity to label nuclear A3B in neoplastic cells and additionally react with cytoplasmic A3G and A3A in tumor-infiltrating lymphocytes (TILs) and tumor-associated monocytes/macrophages, respectively.

### 3.6. Comparisons with Commercial Rabbit mAbs

**Figure 6:**
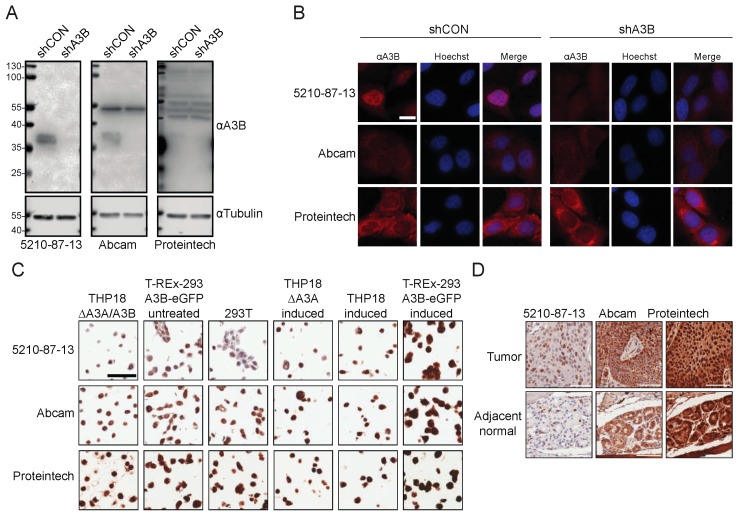
Comparison of 5210-87-13 and commercial rabbit mAbs advertised for A3B: (**A**) Immunoblots of whole cell lysates from U2OS cells expressing a control shRNA (shCON) or shA3B probed with the 5210-87-13, Abcam, or Proteintech mAb with anti-Tubulin as a loading control; (**B**) IF microscopy images of U2OS cells stained with the indicated rabbit mAb (scale bar is 10 µm); (**C**) IHC images of the indicated CMA cell lines stained with the 5210-87-13, Abcam, or Proteintech mAb (scale bar is 60 µm); and (**D**) IHC images of serial FFPE sections from the same tumor or adjacent normal tissue stained with the 5210-87-13, Abcam, or Proteintech mAb (scale bars are 60 µm). See text for details.

Finally, a head-to-head comparison was made with the only two commercial rabbit mAbs advertised for reactivity with human A3B (Abcam 184990 and Proteintech 14559-1-ap). First, immunoblots were done using U2OS cells with endogenous *A3B* (shCON transduced) or *A3B* knockdown (shA3B). As above, the 5210-87-13 mAb showed strong A3B reactivity (Figure 6A, left). The Abcam mAb was able to similarly recognize endogenous A3B, but reacted with an unknown 54 kDa protein (Figure 6A, middle). The Proteintech mAb showed no A3B reactivity and yielded only multiple non-specific bands (Figure 6A, right). Next, the same U2OS cell pools were examined by immunofluorescent microscopy. As above, the 5210-87-13 mAb yielded strong nuclear staining of native A3B, which was diminished by mRNA knockdown (Figure 6B). In contrast, both the Abcam and the Proteintech mAbs demonstrated strong cytoplasmic staining, regardless of endogenous A3B expression levels (Figure 6B). Then, the mini-CMA described above was probed using the three different rabbit mAbs. The 5210-87-13 mAb showed distinct nuclear staining, of moderate to strong intensity, in the cell lines expressing A3B, whereas the other two mAbs generated non-discriminatory, diffuse, and cell-wide staining, regardless of A3B expression levels (Figure 6C). Finally, serial sections of an oral malignant tumor (squamous cell carcinoma) with matched adjacent normal glandular tissue were probed with the three different rabbit mAbs. As expected from the other immunoassay results, these IHC experiments showed that only the 5210-87-13 mAb stained the nuclear compartment of many of the tumor cells, whereas the corresponding matched normal tissue was, overall, negative for A3B (Figure 6D). In contrast, the Abcam reagent demonstrated granular cytoplasmic and paranuclear immunostaining in cancer cells, as well as in adjacent normal glandular epithelial cells (Figure 6D). The Proteintech mAb yielded a non-discriminatory, strong, and diffuse nuclear and cytoplasmic staining of nearly every cell type in the tumor, as well as matching normal specimens (Figure 6D). Each of the head-to-head immunoassays described here was done in parallel with the exact same substrates and reagents, apart from the primary mAbs.

## 4. Discussion

In this study, we report the creation and validation of a rabbit mAb, 5210-87-13, which binds to the human DNA cytosine deaminase A3B. This mAb tested positive in a wide variety of immunoassays, including ELISA, IB, IF, and IHC. As expected from the positive IF results, it was also useful for FLOW (data not shown). Despite our original goal of identifying an A3B-specific mAb, the 5210-87-13 mAb and all other hybridoma candidates still cross-reacted, to varying degrees, with the highly related DNA deaminases A3A and A3G. However, many results from experiments utilizing the 5210-87-13 mAb are still interpretable and, in many instances, definitive, as: (1) A3B was the only one of these enzymes expressed in the system (e.g., transient transfections and many different cancer cell lines and tumor tissues); (2) the three enzymes can be distinguished by kilodalton size and mobility (immunoblots); and (3) the enzymes localize to different subcellular compartments (A3B shows nuclear localization, whereas A3A and A3G are cytoplasmic). The experiments reported here also show that the 5210-87-13 mAb does not cross-react with more distantly related human APOBEC3 family members (i.e., A3C/D/F/H, AID, and APOBEC1).

The 5210-87-13 mAb detailed here has already proven effective in a range of IB experiments. We first used it to study induction of endogenous A3B expression by the PKC/non-canonical NF-κB signal transduction pathway [27]. We subsequently used it in IB experiments to study SIV Vif-mediated degradation of endogenous A3B [69], endogenous A3B upregulation by polyomavirus infection [11,65], A3B lysine post-translational modification [70], and endogenous A3B interaction with EBV BORF2 [16]. The 5210-87-13 mAb has also been shared with colleagues, who have independently demonstrated utility in anti-A3B IB experiments [71,72]. In addition, another independent study used 5210-87-13 to monitor A3A and A3B induction in IB and IF experiments [73]. Thus, the 5210-87-13 mAb detailed here will be helpful for studying human A3B using these and other assays, including IHC.

Importantly, the studies here are the first to demonstrate the utility of the 5210-87-13 mAb in IHC, which is the gold-standard technique for clinical detection of proteins in pathology labs worldwide. IHC efficacy was demonstrated using both a mini-CMA and FFPE tumor tissues. Moreover, commercial rabbit mAbs advertised for A3B failed to experimentally yield the characteristic nuclear staining of this enzyme. Thus, to the best of our knowledge, the 5210-87-13 mAb is the only antibody that is presently useful for IHC studies. This approach opens the door to a wide range of retrospective and prospective clinical studies using 5210-87-13 to assess the diagnostic and/or prognostic utility of nuclear A3B levels in multiple human malignancies, including those of the bladder, cervix, breast, head/neck, lung, and ovary. For instance, as high-risk HPV infection causes A3B upregulation [28,29,74], nuclear IHC staining using 5210-87-13 may be useful as a sentinel biomarker (or surrogate biomarker, in addition to p16) for diagnosing HPV-related precancerous or cancerous lesions. In addition, following initial results in breast cancer using mRNA expression levels [30,33,75], tumors immunoreactive for A3B using 5210-87-13 may elicit higher degrees of evolvability, in comparison to those that are A3B-negative. A3B protein levels may, therefore, provide a predictive biomarker for detrimental clinical outcomes, including tumor drug resistance and metastasis.

## 5. Conclusions

We conclude that the rabbit mAb, 5210-87-13, is useful for a wide variety of immunoassays, including ELISA, IB, IF, FLOW, and IHC. This monoclonal antibody binds human A3B as intended; however, unavoidable cross-reactivity with human A3A and A3G is a technical limitation that may limit or complicate utility in some applications. Nevertheless, the 5210-87-13 mAb will be an excellent reagent for many fundamental studies, and it should also be evaluated for clinical efficacy as a biomarker to help identify A3B-positive tumors for customized treatments.
